# West Nile Virus Outbreak in Houston and Harris County, Texas, USA, 2014

**DOI:** 10.3201/eid2308.170384

**Published:** 2017-08

**Authors:** Diana Martinez, Kristy O. Murray, Martin Reyna, Raouf R. Arafat, Roberto Gorena, Umair A. Shah, Mustapha Debboun

**Affiliations:** Harris County Public Health, Houston, Texas, USA (D. Martinez, M. Reyna, R. Gorena, U.A. Shah, M. Debboun);; Baylor College of Medicine and Texas Children’s Hospital, Houston (K.O. Murray);; Houston Health Department, Houston (R.R. Arafat)

**Keywords:** West Nile virus, vector, vector-borne infections, mosquito, *Culex* *quinquefasciatus* (Say), *Aedes* *albopictus* (Skuse), epidemiology, outbreak, economic impact, attack rates, neuroinvasive, fever, Houston, Harris County, Texas, viruses

## Abstract

Since 2002, West Nile virus (WNV) has been detected every year in Houston and the surrounding Harris County, Texas. In 2014, the largest WNV outbreak to date occurred, comprising 139 cases and causing 2 deaths. Additionally, 1,286 WNV-positive mosquito pools were confirmed, the most reported in a single mosquito season.

West Nile virus (WNV) emerged in the United States in 1999 and in Texas in 2002 (*1*). During 2002–2011, Texas reported 2,202 WNV cases and 135 deaths (*2*). In 2012, the largest statewide WNV outbreak in Texas occurred; 1,868 cases and 89 deaths were reported (*3*).

Every year since 2002, WNV has been detected in Houston and the surrounding Harris County (Houston/Harris County), the third most populous US county. This area was affected by the statewide outbreak in 2012, and then in 2014, the largest WNV outbreak to date occurred in this area. We examined the epidemiology of WNV in Houston/Harris County in 2014.

## The Study

We investigated epidemiologic surveillance data on reported WNV case-patients who had onset of illness during January 1, 2002–December 31, 2014, from the Texas Department of State Health Services and the Houston-area local health departments: Harris County Public Health (HCPH) and the Houston Health Department (HHD). We determined case severity (West Nile neuroinvasive disease [WNND] and West Nile fever [WNF]) by using established criteria (*4*) and calculated attack rates by demographic characteristics by using population estimates for 2014 (*5*). We used Stata 14.0 (StataCorp LLP, College Station, TX, USA) to conduct statistical analyses.

**Figure 1:**
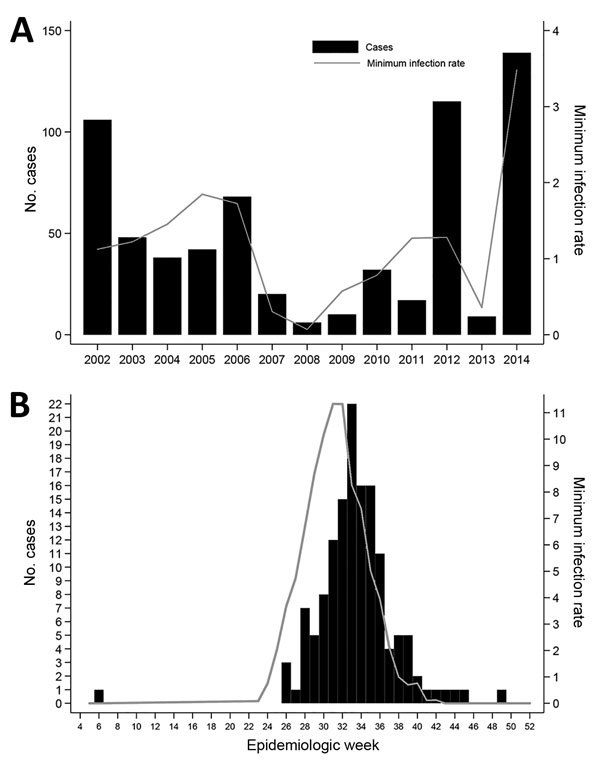
Epidemic curves depicting number of cases of West Nile among humans and minimum infection rate (MIR) of positive mosquito pools by year (A) and by epidemiologic week (B), Houston/Harris County, Texas, 2002–2014. MIR was calculated by the formula (no. positive mosquito pools × 1,000)/no. female mosquitoes pooled).

During 2002–2014, a total of 650 human WNV cases were reported to HCPH and HHD (Technical Appendix Table 1), including 35 deaths (case-fatality rate 5.4%). This total represented 14% of all cases reported from Texas during this period. The epidemic curve revealed an increased number of cases in 2002, 2006, 2012, and 2014 (Figure 1), which correlated with the minimum infection rate for mosquitoes (Pearson correlation coefficient r = 0.74).

During 2014, Harris County reported its highest number of WNV human cases to date: 139 cases and 2 deaths. This total represented 37% of all cases in Texas. Most (76%) cases were WNND (Table 1). Overall, incidence was 3.26 cases/100,000 population; 69% of case-patients were male. Mean case-patient age was 57 years (range 6–92 years); most (76%) case-patients were ≥45 years of age. Attack rates increased with age, from 0.4 cases/100,000 population for those <18 years of age to 14.5 cases/100,000 population for those ≥65 years of age. A higher attack rate was observed among non-Hispanic whites when compared with black, Hispanic white, and Asian populations. We did not detect statistically significant differences between WNND and WNF occurrence related to race/ethnicity, age, or sex (all p values >0.05). This finding is in contrast to the Texas 2012 statewide outbreak, during which being male, >65 years of age, and of a minority population were statistically associated with WNND (*3*). Although results from this study had similar trends, the lack of statistical significance was likely caused by the small sample size of WNF cases (n = 34).

**Table 1:** Demographics and attack rates of West Nile virus cases reported to HCPH and HHD, Houston/Harris County, Texas, USA, 2014*

Case-patient characteristics	All case-patients, no. (%), n = 139	Attack rate†/100,000 population	WNF, no. (%), n = 34	WNND, no. (%), n = 105
Sex				
M	96 (69)	4.5	26 (76)	70 (67)
F	43 (31)	2.0	8 (24)	35 (33)
Age, y				
˂18	5 (4)	0.4	1 (3)	4 (4)
18–24	3 (2)	0.7	1 (3)	2 (2)
25–44	25 (18)	1.9	6 (18)	19 (18)
45–64	52 (37)	5.2	11 (32)	41 (39)
˃65	54 (39)	14.5	15 (44)	39 (37)
Race/ethnicity				
White, non-Hispanic	73 (53)	5.3	17 (50)	56 (53)
Black	19 (14)	2.4	6 (18)	13 (12)
White, Hispanic	30 (22)	1.7	4 (12)	26 (25)
Asian	2 (1)	0.7	1 (3)	1 (1)
Other/unknown	15 (11)	NA	6 (18)	9 (9)

*HCPH, Harris County Public Health; HHD, Houston Health Department; WNF, West Nile fever; WNND, West Nile neuroinvasive disease; NA, not applicable. †Attack rates based on 2014 population estimates from the Texas State Data Center (*3*), accessed February 10, 2017. Total population of Harris County = 4,269,608.

In 2014, the HCPH Mosquito and Vector Control Division also identified the largest number of WNV-positive mosquito pools to date: 1,286 positive pools were identified through the Rapid Analyte Measurement Platform (Response Biomedical Corp, Burnaby, British Columbia, Canada) (Table 2). In Harris County, 782,551 mosquitoes were collected, comprising 34 of 56 species recorded (*6*). We tested 12,608 mosquito pools from 19 species (Technical Appendix Table 2) and confirmed in 1,285 *Culex quinquefasciatus* (Say) mosquito pools and in 1 *Aedes albopictus* (Skuse) pool. The first WNV-positive mosquito pool was detected during epidemiologic week 23 of 2014 (June 6). Viral detection continued for 20 weeks, ending in week 42 (October 14). The peak of positive pools (n = 168) occurred during week 30 (July 20–26). In comparison, dates of symptom onset for human cases occurred during February 5–December 1, 2014, and peaked during week 33 (Figure 1).

**Table 2:** WNV-confirmed mosquito pools collected from 4 different types of mosquito traps, Houston/Harris County, Texas, USA, 2014*

Trap type†	Species	No. traps	Total no. pools	Females pooled	WNV	SLE	WNV MIR
CDC Gravid Trap	*Culex quinquefasciatus*	3,971	5,025	192,441	716	0	3.72
CDC Miniature Light Trap	*Cx. quinquefasciatus*	3,748	4,617	154,124	566	1	3.67
Biogents Sentinel Trap	*Cx. quinquefasciatus*	926	54	680	3	0	4.41
CDC Gravid Trap	*Aedes albopictus*	1,315	359	952	1	0	1.05

*WNV, West Nile virus; SLE, St. Louis encephalitis virus; MIR, minimum infection rate. †CDC Gravid Trap and CDC Miniature Light Trap, John W. Hock Company, Gainesville, FL; Biogents Sentinel Trap, Biogents AG, Regensburg, Germany.

**Figure 2:**
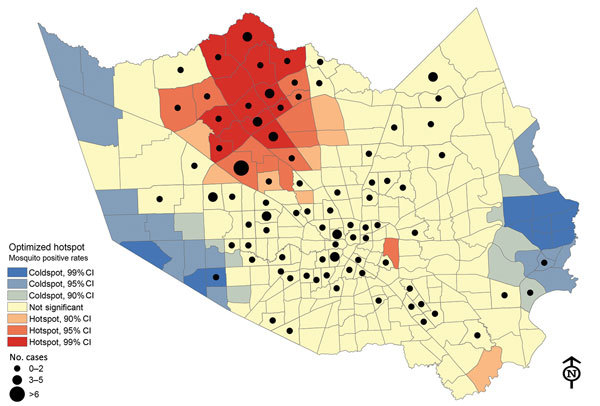
Optimized hotspot analysis results showing residential locations of persons who had West Nile virus and their association with positive mosquito hotspots, Houston/Harris County, Texas, 2002–2014. Red “hot” areas represent statistically significant high-risk virus-positive mosquito activity, compared with blue “cold” areas with low risk for positive mosquitoes.

We used ArcMap 10.2.1 (Environmental Systems Research Institute, Inc. [ESRI], Redlands, CA, USA) to determine the distributions of positive mosquitoes and residences of case-patients where available (n = 128). Throughout the transmission season, WNV was detected in 237 (88%) of the 268 Mosquito Control Districts (MCD) in Harris County. We used the Optimized Hot Spot Analysis Tool (ArcGIS Pro; ESRI, Redlands, CA, USA) to calculate the Getis-Ord Gi statistic, which we used to determine statistically significant clusters (hotspots) of mosquito activity. We then converted data for case-patients to graduated frequency dots to determine spatial patterns. In the hotspot analysis (Figure 2), red shading indicates MCDs with statistically significant clustering of positive mosquito pools (90%, 95%, and 99% CIs) compared with neighboring MCDs. The dark blue areas show where mosquito-positive pools were statistically less likely to occur. We observed a similar hotspot pattern in prior years (D. Martinez et al., Harris County Public Health, Houston, TX, unpub. data). Hotspots are likely related to ecologic areas with higher vegetation and creeks (*7*).

The 2014 WNV outbreak in Houston/Harris County was unexpected, particularly because transmission activity across the state was at low levels, and only 379 cases were reported statewide. It is likely that the actual number of WNV cases was considerably higher, particularly considering that 76% of reported case-patients had severe WNND, which indicates a probable diagnostic bias. Prior studies showed that <1% of WNV infections manifest as WNND (*8*,*9*). A recent study in Houston showed that WNV testing was ordered for only 37% of viral meningitis/encephalitis case-patients (*10*), further highlighting concerns for underestimation of the true extent of disease.

We recently reported that WNV activity appears to have a 3-year pattern that showed increases in reported cases observed in 2003, 2006, 2009, and 2012 (*2*,*3*). During the years in which we observed increased numbers of cases, we also observed earlier detection of positive mosquito pools, which first appeared in May, compared to June in non-outbreak years. Vector abundance and increased amplitude of transmission are likely driven by ecologic and environmental factors. In 2014, we were unsure why such a large outbreak occurred in this region compared with prior years. Weather patterns during 2012–2014 were different: 2012, after the drought of 2011, had mild winter temperatures. Conversely, 2014 had below-average winter temperatures, above-average rainfall, and no drought conditions. Further research would aid our understanding of factors that drive WNV transmission.

Vector surveillance is vital to disease prevention. The year-round Integrated Mosquito Management Program includes surveillance-guided vector control. It is difficult to estimate the number of WNV cases prevented; however, considering the high minimum infection rate in 2014 in the densely populated city of Houston, we believe that 139 cases are a much lower number than what could have otherwise occurred if no surveillance/control activities had been implemented. Barber et al. found that vector control is cost-effective if at least 15 cases of WNND are prevented (*11*).

Continuous WNV disease activity has had a high economic effect on the Houston/Harris County area. By using estimates provided by Barber et al. (*11*) (adjusted to 2014 US dollar value [*12*]), we calculated the acute medical care and productivity costs of the 2014 outbreak to be ≈$6 million (Technical Appendix Table 3). In addition to high medical costs, costs related to rehabilitation/long-term care, surveillance, and vector control would be considerable.

## Conclusions

The 2014 outbreak of WNV in the Houston/Harris County area is a reminder of the continuous effect and the unpredictable nature of disease transmission. With no specific therapeutic options or vaccine available, the costs related to medical care, surveillance, and vector control will continue to mount. Public health authorities should remain vigilant to prevent mosquitoborne infections. We expect endemic levels of transmission and occasional epizootics in the years to come.

Technical Appendix West Nile virus (WNV) 2002–2014 case counts and outcomes, WNV and St. Louis encephalitis–positive mosquitoes during 2014, and estimated costs of acute medical care and loss of productivity related to WNV during 2014 in Houston and Harris County, Texas.
