# Supplementary material for: West Nile Virus Outbreak in Houston and Harris County, Texas, USA, 2014
Source: Emerg Infect Dis. 2017 Aug;23(8):1372–6. doi: 10.3201/eid2308.170384 (PMC5547786; doi:10.3201/eid2308.170384)
Supplement: Technical Appendix — West Nile virus (WNV) 2002–2014 case counts and outcomes, WNV and St. Louis encephalitis–positive mosquitoes during 2014, and estimated costs of acute medical care and loss of productivity related to WNV during 2014 in Houston and Harris County, Texas. [file 17-0384-Techapp-s1.pdf]

Article DOI: https://doi.org/10.3201/eid2308.170384

West Nile Virus Outbreak in Houston and Harris County, Texas, USA, 2014

Technical Appendix

Technical Appendix Table 1. Total number of WNV cases by year for the state of Texas and Houston/Harris County, TX*

	Texas WNV cases			Harris County, Texas WNV cases
Year	Fever	Neuroinvasive	Total deaths	Total deaths
2002	NR	202	202 (13)	106 (12)
2003	289	431	720 (37)	48 (3)
2004	57	119	176 (8)	38 (3)
2005	67	128	195 (11)	42 (1)
2006	121	233	354 (32)	68 (5)
2007	90	170	260 (16)	20 (2)
2008	24	40	64 (1)	6 (1)
2009	22	93	115 (9)	10 (1)
2010	12	77	89 (6)	32 (1)
2011	7	20	27 (2)	17 (0)
2012	1,024	844	1,868 (89)	115 (4)
2013	70	113	183 (13)	9 (0)
2014	126	253	379 (6)	139 (2)
Total	1,909	2,723	4,632 (243)	650 (35)

*WNV, West Nile virus; NR, none reported.

Technical Appendix Table 2. Number of WNV and SLE confirmed positive mosquito pools collected from different mosquito species throughout Harris County and the City of Houston, TX, 2014

Mosquito species	No. traps	Total no. females	Total no. males	Percent collected	Total no. pools	Females pooled	WNV+	SLE+
Culex quinquefasciatus	8,651	720,121	93,115	92.02	9,698	347,259	1,285	1
Aedes taeniorhynchus	564	18,592	4,833	2.37	30	205	0	0
Aedes albopictus	2,449	17,776	10,940	2.27	1,208	13,737	1	0
Aedes aegypti	1,957	7,876	3,062	1.00	1,114	6,375	0	0
Culex salinarius	1,361	7,498	147	0.95	277	998	0	0
Culex restuans	645	2,452	55	0.31	42	89	0	0
Culex coronator	613	2,019	108	0.25	67	200	0	0
Aedes vexans	402	1,685	53	0.21	22	27	0	0
Culex nigripalpus	530	1,042	78	0.13	101	186	0	0
Psorophora columbiae	164	701	139	0.08	2	2	0	0
Culiseta inornata	190	643	20	0.08	4	4	0	0
Culex erraticus	156	472	136	0.0603	19	30	0	0
Anopheles quadrimaculatus	260	403	85	0.0514	9	12	0	0
Psorophora cyanescens	29	370	3	0.0472	1	1	0	0
Aedes sollicitans	86	307	9	0.0392	1	1	0	0
Psorophora howardii	32	143	23	0.0182	0	0	0	0
Psorophora ferox	36	61	13	0.0077	3	3	0	0
Psorophora longipalpus	23	50	1	0.0063	0	0	0	0
Mansonia titillans	33	46	0	0.0058	4	6	0	0
Anopheles crucians	35	46	1	0.0058	4	4	0	0
Uranotaenia lowii	38	43	1	0.0054	0	0	0	0
Toxorhynchites rutilus septentrionalis	33	32	4	0.0040	0	0	0	0
Culex tarsalis	17	30	1	0.0038	0	0	0	0
Psorophora ciliate	16	28	0	0.0035	0	0	0	0
Aedes triseriatus	25	28	0	0.0035	2	2	0	0
Uranotaenia sapphirina	23	23	1	0.0029	0	0	0	0
Anopheles punctipennis	14	16	1	0.0020	0	0	0	0
Anopheles pseudopunctipennis	4	12	1	0.0015	0	0	0	0
Aedes fulvus pallens	9	11	1	0.0014	0	0	0	0
Psorophora horrida	3	8	3	0.0010	0	0	0	0
Orthopodomyia signifera	7	7	0	0.0008	0	0	0	0

Mosquito species	No. traps	Total no. females	Total no. males	Percent collected	Total no. pools	Females pooled	WNV+	SLE+
Aedes nigromaculis	4	5	0	0.0006	0	0	0	0
Aedes atlanticus	3	4	0	0.0005	0	0	0	0
Coquillettidia perturbans	1	1	0	0.0001	0	0	0	0

*WNV, West nile virus; SLE, St. Louis encephalitis virus; TX, Texas.

Technical Appendix Table 3. Calculations of the cost of acute medical care and loss of productivity as a result of the 2014 Houston/Harris County, Texas WNV outbreak*

Case status	Barber *et al*. estimated costs per case in 2005 (range)	Cost adjusted to 2014 US dollars† (range)	No. cases	Cost estimate of 2014 outbreak
WNND	$46,531 ($13,201-$140,257)	$56,403 ($16,001-$170,014)	105	$5,922,315 ($1,680,105-$17,851,470)
WNF	$1,170 ($1,128-$1,235)	$1,418 ($1,367-$1,497)	34	$48,212 ($46,478–$50,286)
Total	NA	NA	139	$5,970,527 ($1,726,583–$17,901,756)

*WNF, West Nile Fever; US, United States; WNND, West Nile neuroinvasive disease; NA, not applicable.
†Bureau of Labor Statistics Consumer Price Index Calculator: www.bls.gov/data/inflation_calculator.htm
